# The applicability and efficacy of *Micro-Video Psychological Training Camp* in groups with mild to moderate symptoms of depression and anxiety: A prospective and randomized controlled trial protocol

**DOI:** 10.3389/fpsyt.2022.991465

**Published:** 2023-01-17

**Authors:** Wenqing Zhao, Shuangyi Chen, Jun Hu, Qing Zhou, Jing Tao, Rui Gao, Jie Zhang, Shanshan Su, Yuan Wang, Yousong Su, Yihua Peng, Yanru Wu, Qing Fan, Weibo Zhang, Wenhui Jiang, Jun Cai, Jianyin Qiu

**Affiliations:** ^1^Shanghai Mental Health Center, Shanghai Jiao Tong University School of Medicine, Shanghai, China; ^2^Shanghai Xuhui Mental Health Center, Shanghai, China

**Keywords:** depression and anxiety symptoms, mental resilience, self-service, psychological intervention, randomized controlled trial (RCT), protocol

## Abstract

**Background:**

Mental health is a global issue requiring global attention. Depression and anxiety are two of the most common mental disorders (CMDs) and are characterized by high incidence and high comorbidity. In recent years, the prolonged COVID-19 pandemic and exacerbated social instability have posed significant challenges to the mental resilience and mental health outcomes of the global population. Now more than ever, with an increase in mental health needs, it has become even more crucial to find an effective solution to provide universal mental healthcare. Psychotherapy is of vital importance for those coping with symptoms of depression and anxiety and is used to enhance mental resilience. However, such therapy can be difficult to access in reality. In this context, the *Micro-Video Psychological Training Camp (MVPTC)* platform will be developed.

**Objectives:**

As an online self-help platform for psychological intervention, the *MVPTC* platform was developed for those who suffer from mild to moderate symptoms of depression and/or anxiety and is tasked with the goal of reducing depressive and anxious symptoms while improving mental resilience. Thus, this study will be carried out to verify its efficacy and applicability.

**Methods:**

In this parallel-group, randomized controlled trial, a total of 200 mild to moderately depressed and/or anxious adults seeking self-help will be randomly recruited and assigned to either the micro-video psychological intervention group or the wait list control group. Online measurements by self-assessment will be taken at baseline, post-intervention, 1-month, and 3-month follow-up.

**Results:**

The primary results will involve symptoms of depression and anxiety. The secondary results will involve mental resilience. An analysis will be conducted based on the intention-to-treat principle.

**Discussion:**

This trial will examine whether the *MVPTC* platform for the relief of symptoms and the enhancement of resilience in a population screened for depression and anxiety symptoms proves effective and applicable. Large-scale resilience enhancement may benefit public mental health in terms of preventive interventions, managing depressive and anxiety symptoms, and promoting mental health. With the *MVPTC*-based method being applied, a brief, efficient, and structured intervention model can potentially be established, having the potential to provide necessary and accessible mental support for an extensive target group.

**Clinical trial registration:**

http://www.chictr.org.cn/, identifier ChiCTR2100043725.

## 1. Introduction

### 1.1. Background

Mental health is a global issue requiring global attention. Depression and anxiety disorder, the two main diagnostic categories of common mental disorder (CMD), are prevalent in many populations, with symptoms ranging from mild to severe. According to estimates, there are over 300 million people (making up 4.4% of the global population) who suffer from depression, and over 264 million people (equivalent to 3.6% of the world’s population) who are affected by an anxiety disorder, with a large proportion of the world’s population suffering from both (comorbidity) (1). Social functioning and quality of life may be affected to varying degrees from the onset of prodromal symptoms to a definite diagnosis (2–4), concomitant with personal stress, and social burden. China is one of the countries with the highest burden of depression and anxiety in the world (5).

To some extent, depression and anxiety are considered stressor-related diseases (6). In low- and middle-income countries (LMICs), people can be more easily affected by various stressors, making them more vulnerable to developing psychological symptoms and/or mental disorders, including depression and anxiety (7, 8). In terms of defining psychological resilience, this refers to the capacity of individuals to cope with challenges or stress while maintaining mental health despite exposure to a myriad of adversity in life (6, 9). Resilience can also be considered a significant factor in protecting people from developing symptoms of mental disorders in emergency situations (2, 10). Meanwhile, studies have also shown that resilience interventions can improve depressive symptoms in patients with depression (11). It can be seen that resilience intervention plays a key role in the development of depression and anxiety symptoms, from the prodromal stage with subclinical symptoms to moderate mental distress in patients with a definite diagnosis.

In recent years, the prolonged COVID-19 pandemic and waning economies, which have aggravated short- and long-term stresses and increased social instability, have posed significant challenges to the mental resilience and mental health outcomes of the global population, especially for susceptible groups of the population (12). Some studies report that the number of people with depression and anxiety is on the rise (13).

As an effective and evidence-based treatment protocol, psychotherapy is of great importance in the management of anxiety and depression (14) and is also a factor that can enhance resilience, as suggested by previous studies. Enhanced self-esteem and increased self-efficacy, emotional regulation, meaning-making, flexible coping capacity, and better interpersonal relationships are the main preventative factors associated with resilience. All of this can be improved by psychological intervention (15), thereby relieving the symptoms of mental disorders and improving mental health (11, 16).

Some effective evidence-based therapies currently exist, but they are not easily accessible, much less in terms of access to standard and effective treatment procedures. Larger populations have a greater need for mental health services, but the real number of patients who can access effective interventions remains limited in number (7, 8). Studies have suggested that over 70% of patients with depression do not seek treatment (17, 18). In fact, among the group of patients who have received psychotherapy, only 27% of them received standardized treatments (19). The demand for interventions involving mental health among patients is low and is the result of various obstacles of differing degrees. Apart from geographic distance and outbreak isolation, the stigma attached to mental disorders remains one of the more prominent obstacles (20). Even when patients are ready to seek treatment, they may still encounter difficulties, such as a shortage of reliable and quality services, a limited number of qualified and professional service providers, and prolonged wait times, as these commonly occur in underdeveloped or isolated areas (21, 22).

Now more than ever, with an increase in mental health needs, it becomes even more vital to find an effective solution that can afford people universal mental healthcare (4). Therefore, a therapeutic tool combining new Internet technology and psychological intervention [e.g., Internet-Based Cognitive Behavior Therapy (iCBT) and Computerized Cognitive Behavior Therapy (cCBT)] will be developed to effectively address this issue. Some studies have reported that digital self-service psychological intervention has an active treatment effect on depression (23, 24) as well as anxiety disorders (25–27). As one of the earliest groups studying intelligent psychological intervention in China, our team already has a foundation in computerized cognitive behavioral therapy for obsessive-compulsive disorder and substance-use disorders (28–30) and has developed an artificial intelligence (AI)-based psychological Chatbot app to help with anxiety disorders.

In this study, the *Micro-Video Psychological Training Camp (MVPTC)*, a self-service online psychological intervention platform, will be developed and aims to be more accessible to a larger number of people. This intervention method, as a supplement to existing psychotherapy resources, can monitor mood, improve mental resilience, and provide psychotherapy based on cognitive behavior therapy (CBT), incorporating core techniques and principles of other psychological therapy methods. For participants with mild to moderate depression and/or anxiety with no immediate risk, treatment indication can be used to alleviate disease symptoms. Furthermore, as an indicated prevention or early treatment intervention, this method has the potential to reduce the risk of disease in the future among ordinary people or high-risk groups, realizing preventive intervention for more vulnerable groups (4).

### 1.2. Objectives

In this study, a prospective, parallel-group, randomized controlled clinical trial will be conducted to probe the applicability and efficacy of *MVPTC* on alleviating symptoms and improving mental resilience in participants with mild to moderate depression and/or anxiety.

Specifically, this trial will be designed to see if the symptoms of depression and/or anxiety are significantly reduced and mental resilience is significantly improved in the micro-video psychological intervention group when compared with the wait list control group.

## 2. Methods

### 2.1. Trial design

This will be a single-center, parallel-group, randomized controlled trial. A total of 200 participants will be randomly chosen and equally divided into the micro-video psychological intervention group and the wait list control group based on a randomly generated table of numbers with 100 participants in each group. The self-service psychological intervention group will be the testing group, and the wait list control group will be the control group.

### 2.2. Setting and recruitment

**FIGURE 1:**
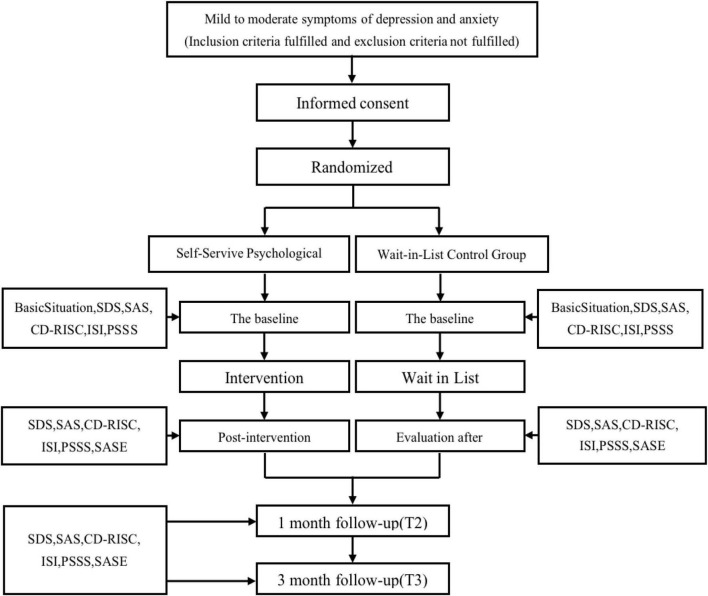
Study design and measurement time points. SDS, The Self-Rating Depression Scale; SAS, The Self-Rating Anxiety Scale; CD-RISC, The Connor-Davidson Resilience Scale; ISI, Insomnia Severity Index; PSSS, Perceived Social Support Scale; SASE, Self-Assessment Scale of the Overall Efficacy and Satisfaction of Participants.

Mild to moderately depressed and anxious adults seeking self-help will be recruited from the psychological clinic of the SMHC, the community, and the Internet. Those who will be involved in recruitment and administration, i.e., the referring doctor (outpatient psychiatrists in SMHC), community staff (psychiatrists in the community center), and mailbox manager (psychotherapists certified by the National Health Commission), are all familiar with our study. They will conduct an initial face-to-face or telephone screening for about 15 min and then recommend suitable participants to our trained researchers. Next, our researchers will make a detailed introduction to these participants and extend a participation invitation for the trial. Before conducting the screening visit, voluntary participants in the trial will sign the electronicinformed consent forms (ICFs). During the screening visit, we used the Self-Rating Depression Scale (SDS) and the Self-Rating Anxiety Scale (SAS) scales as a criterion for inclusion access and the Mini International Neuropsychiatry Interview (MINI 6.0.0) as a diagnostic assessment to exclude other psychiatric disorders that interfered with the study. Moreover, significant suicidal ideation was included in the screening visit (i.e., they had active thoughts, plans, or behaviors of self-injury or suicide in recent months or in the past). Other criteria are listed in the “participants and eligibility” section. Participants who meet the criteria will be able to proceed to the next stage. The specific flowchart is found in Figure 1.

### 2.3. Participants and eligibility

Participants will be included in this study if (1) they are 18–55 years of age; (2) they have mild to moderate depressive symptoms with a score between 53 and 72 on the SDS, mild to moderate symptoms of anxiety with a score between 50 and 69 on the SAS, or both; (3) they have a junior/middle school education or above and are good with using a smartphone; (4) they have not received psychiatric medication; (5) they have not received systematic psychotherapy within the past 6 months but are relatively motivated to receive treatment; and (6) they volunteered to participate and signed the ICF after fully understanding all aspects of the trial.

Participants will be excluded from the study if they meet the following criteria. (1) They have an onset of a concurrent severe physical disease that is not well controlled or if any existing physical disease that requires priority for physical treatment is exacerbated. (2) They have an immediate risk or medical treatment indication, such as depressive symptoms or anxiety symptoms that exceed the threshold limit or significant suicidal ideation reflected on the initial screening visit and scale (initial SDS item 20 score > 1). Those who fail to meet the selection criteria will be notified and advised to seek the help of a psychiatrist at an outpatient clinic. In cases of intense suicidal ideation, people will be urgently referred to a psychiatrist for a higher level and more urgent treatment. (3) They suffer a lifetime DSM-5 psychiatric disorder, other than depression and anxiety disorder, which includes psychotic symptoms, neurological disease, substance abuse, personality disorder, and mental retardation. (4) They have participated in other clinical studies.

### 2.4. Randomization

Randomization of the trial will be based on a computer-generated randomization list. After returning their ICF and completing the baseline questionnaire, people who meet the inclusion criteria will be randomly allocated to the *MVPTC* intervention group or to the waiting list control group and then will receive a notification *via* WeChat message. Later, a computer program will allocate participants using a generated randomization list. Block randomization in blocks of two will be used to ensure an equal distribution of participants across conditions.

### 2.5. Intervention

#### 2.5.1. Medication

During the baseline screening phase, all participants will not receive any psychiatric medication as there is no immediate risk and treatment indication. Participants were tracked for the medication during the study. They are not restricted in their medication and have access to regular help in this process. If participants take medication during the study, a record will be kept.

#### 2.5.2. Wait list control group

Participants in this control group will be on a waiting list for 4 months. Afterward, they will receive the self-help micro-video psychological intervention after the study. A waiting list control group is ethically acceptable given that there is no immediate risk or indication for medical treatment (31). At the same time, all members of the wait-in-list will also be screened for regular monitoring and preparedness for emergency intervention protocols and referrals to clinical services as necessary. During this time, they will have unrestricted access to professional help if necessary. The only difference with the intervention group is whether self-help methods are actively taught.

#### 2.5.3. Micro-video psychological intervention group

##### 2.5.3.1. Technical overview and customization

After random assignment, the participants in the micro-video psychological intervention group will enter the *MVPTC* and take eight group sessions of micro-video intervention every 3 days. *MVPTC* is an online, self-service smartphone applet with assessment and intervention functions and content such as mindfulness audio recordings and articles on psychoeducation. Many additional functions are under development. The virtual psychotherapist, Xiao Wei, as named by the SMHC, is the training’s virtual guide. The word “Wei” in the name “Xiao Wei” in Chinese denotes tenderness, support, and simplicity, representing that Xiao Wei will guide the participants when they are depressed and anxious in a simplified and supportive form of psychotherapy.

*Micro-Video Psychological Training Camp* is a micro-video self-service psychological intervention platform on WeChat applets. The goal of this platform is to address the current issue of limited psychotherapy resources, rather than replace psychotherapy. After designing the knowledge structure and picking content for the *MVPTC*, psychiatric and psychological professionals at SMHC delve deeper into the platform and its development by reading up on related literature involving the psychological treatment of depression and anxiety. During this period, they frequently scheduled meetings to discuss the outline, compose and modify its script, make animations, and develop its program. After completing preliminary development, user experience tests and interviews with participants were carried out. Later, the intervention content and applet were further improved based on the feedback. Finally, the final launch was completed within a year and is continually improved and upgraded.

On WeChat, the most frequently used app in China, the *MVPTC* applet platform is easily accessible to users. Participants can use the platform conveniently in the privacy of their own homes, with more flexibility, and in a relatively anonymous way. Mandatory hospital or treatment room visitations can be reduced after using this platform, and it brings a lot of convenience for participants even in this pandemic era, no matter where they are. This can save transportation time and streamline the treatment process. In addition, the platform’s online application can reduce the time spent with clinicians while minimizing costs, which can make intervention treatment accessible to more populations.

By using the management system of the applet, all participants can be monitored and tracked by the researchers. While the platform does not have a crisis management function, when participants in both groups face the risk of self-injury, suicide, or exacerbating their symptoms of depression and anxiety, researchers still have the option of halting the study after discussed with the research team. Furthermore, participants in this situation can be recommended by researchers to receive emergency medical treatment. If necessary, the participant’s guardian or family member can be contacted to talk about the possible need for higher-level treatment, such as medication or hospitalization. All of this will be informed consent at the beginning of the study.

##### 2.5.3.2. Integrative support approach

After logging into the WeChat applet and completing the registration, participants will receive a brief welcome message that invites them to participate in the intervention treatment. Here, they will be provided with the necessary information that overviews the intervention form, intervention module, operation, and functions of the intervention treatment. After finishing the opening message, participants can complete the intervention more smoothly.

*Micro-Video Psychological Training Camp* delivering therapy is primarily grounded on the core techniques and principles of CBT (32, 33), integrating psychoeducation, mindfulness (34–36), dialectical behavior therapy (37), as well as interpersonal psychotherapy (38), all of which have proven efficacy in empirical studies on emotional issues including depression, anxiety, and mental resilience. By properly regulating various aspects in terms of emotional, cognitive, behavioral, and interpersonal functions, this therapy can effectively alleviate the effects of depression and anxiety while building up mental resilience. Guided by the virtual therapist Xiao Wei, the therapy content is presented in the form of vivid animation by combining visuals and audio. The duration of these micro-videos in each section ranges from 3 to 5 min and can be repeatedly watched. There is one module every 3 days, for a total of eight treatment modules. Moreover, relevant tasks or homework will be assigned at the final stage of each session. Here, participants can complete them on a self-help basis. A brief review of the session content will also be provided so that participants can apply their knowledge in a flexible and accurate manner. There will also be some optional supplemental resources available on the platform, such as mindfulness audio recordings, which can be downloaded at will.

**FIGURE 2:**
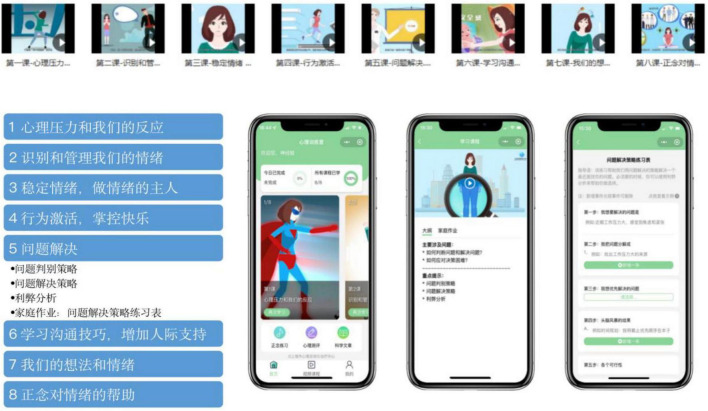
Screenshot startpage, sessions, and homework.

The eight treatment modules are listed as follows: one of the modules is about psychoeducation involving stress, emotions, and our reactions to them; two of the modules are on emotional regulation and involve the identification and management of our emotions, the stabilization of our emotions, and how to be the master of our emotions; one of the modules is on behavior activation; one deals with problem-solving; one concerns communication skills and interpersonal support; one is related to cognitive reconstruction; and one of the modules is concerned with mindfulness. The screenshot startpage, sessions, and homework are shown in Figure 2.

A total of eight specific treatment modules are recommended for each participant in a specific order. They can keep returning to the system and writing down what they have practiced every day, which can be viewed again after recording. This method can enhance user engagement in the homework feedback and interactions, affording participants the chance to apply the techniques they have learned in everyday life, thereby bolstering and consolidating the effects of the treatment protocol.

To encourage participants to continue to study and practice online, the camp gives participants automatic reminders and notification messages in a compassionate and supportive way. Some examples of the sample content are as follows: “(nickname), hello! Today you can start the 6th training session on the “*Micro-Video Psychological Training Camp*” applet to learn about communication skills to help improve your interpersonal support. Please log in to the applet in time to complete this training. I hope to see you make progress!” When participants complete a module, the reminders “You did an awesome job” and “Keep it up” will be displayed on the screen.

### 2.6. Participant timeline

At baseline and the final stage of intervention, participants are required to receive a psychological assessment and a symptom evaluation. Following the 24-day treatment, 1- and 3-month follow-up assessments will also need to be conducted (Table 1). All the evaluation results will be reported to the participants and made into a line chart so they can directly observe the changes in their symptoms. In addition, the line chart can be viewed by other participants at the training camp, which can encourage their proactive participation.

**TABLE 1:** Preliminary experimental results (*N* = 24).

	Before the intervention (M ± SD)	After the intervention (M ± SD)
SDS	61.41 ± 4.92	49.63 ± 10.11
SAS	50.89 ± 7.05	42.40 ± 5.86

### 2.7. Sample size

The results of the pre-experiment were used as a reference for the estimation of the sample size. The preliminary results of the intervention group before and after the intervention in the pre-experiment are shown in Table 1.

From previous studies (39), we know that there was no significant statistical difference between the result of the waiting list control group’s score before and after. Thus, we used the score before the intervention as the outcome score of the waiting list control group and the score after the intervention as the outcome score of the intervention group to calculate the sample size that was based on the two-sample mean to improve the efficiency of the experimental design (40) in the calculation formula when computing the sample size.


(1) $$\eta_{A}=\kappa \eta_{B}\text{⁢and⁢}\eta_{B}=(1+\frac{1}{k}\,){\left(\sigma \frac{z_{1-\alpha }+z_{1-\beta }}{\mu_{A}-\mu_{B}-\delta }\right)}^{2}$$

κ represents the ratio of two groups of sample size. In this study, κ = *1*. σ stands for standard deviation. In this study, the standard deviation before intervention was considered the population standard deviation. *Z1*-α represents the value of the standard normal distribution function corresponding to the level of class I error (test level). *Z1*-β represents the value of the standard normal distribution function corresponding to the level of class II error (test level); Delta represents the boundary between the two sets of values.

The test level α = 0.05 and the degree of certainty β = 0.20 were set. The combined standard deviation of SDS and SAS as well as the boundary values of different outcome indices (the difference between indices considered to be meaningful professionally) were used to calculate the sample size, as the results are shown in Table 2. In light of feasibility, funding, and other practical factors, the study selected 100 cases in each group, or 200 cases in total, as the sample size.

**TABLE 2:** Minimum sample size of each group required to find differences between groups at different threshold levels.

Outcome indicators	Outcome score of intervention group	Outcome score of control group	Combined standard deviation σ	Boundary value δ	Sample size	Loss rate 30%
SDS	50	61	8	5	22	33
				6	32	43
				7	50	61
				8	88	98
				9	198	209
SAS	42	51	7	4	25	36
				5	38	49
				6	68	79
				7	152	163

### 2.8. Discontinuation

If the below conditions are met, participants will be able to choose to withdraw or will be required to quit the study:

a. Somatic disease progression.
b. Serious adverse events.
c. Uncontrollable concurrent disease.
d. Whenever the participant wishes to withdraw.
e. Poor compliance.
f. Instances in which withdrawal from the study can bring more benefits to the participants, including adverse health factors or an increased risk of suicide.

Participants should be clearly informed of their right to withdraw from the study at any time, regardless of their stage of evaluation, treatment, or follow-up. The principal investigator is responsible for the decision to withdraw a participant from the study after consulting other members of the team.

## 3. Outcome measurements and instruments

The study assessment will be completed online by the subjects on a self-help basis. Self-rating scales were used in this study. The analysis of these scales will be conducted by a statistician without knowing how the subjects are allocated.

The efficacy indicators include (1) the primary outcome: the change in the SDS score and the SAS score between groups at different times. In particular, if the reduction rate is ≥50%, an obvious effect will be shown; if the rate is <50 and ≥25%, the effect is only average; and if the rate is <25%, no clinical effect will exist. (2) The secondary efficacy outcome is based on The Connor-Davidson Resilience Scale (CD-RISC) score, which can be increased as symptoms of anxiety and depression improve during the treatment process. In addition, measurements including the Insomnia Severity Index (ISI) and the Perceived Social Support Scale (PSSS) will be provided.

The applicability indicators include the intervention completion rate, the dropping rate, and the Self-Assessment Scale of the Overall Efficacy and Satisfaction of Participants (SASE) score.

### 3.1. Primary outcome measurements

The SDS (41) and the SAS (42) are two common self-rating depression and anxiety scales, respectively, which can play a significant role in measuring the severity of symptoms of depression and anxiety in adults. These two scales include 20 items, and a four-point system (from 0 to 4) is applied for most items. As a clinical instrument, it has outstanding reliability and validity.

### 3.2. Secondary outcome measurements

The CD-RISC (9) has been shown to be a valid and reliable clinical instrument that can measure individual mental resilience levels. There are 25 items that can be divided into three subscales (i.e., toughness, strength, and optimism). These subscales use a five-point system, with each score ranging from 0 (almost never) to 5 (almost always).

### 3.3. Other psychological scales

The ISI (43) is a five-point Likert-type scale with a 7-item self-report that assesses one’s subjective perception of insomnia over the previous week. With high test–retest reliability and internal consistency, the ISI will be implemented for evaluations in this study.

The PSSS (44) consists of 12 items that are designed to evaluate overall perceived social support. The score of the Chinese version of the PSSS, rated from 1 (very false for me) to 7 (very true for me), will be employed. It consists of three dimensions, namely, family support, friend support, and other support.

The SASE is a self-designed questionnaire with eight questions. The scores of the first seven questions are rated from 1 (strongly disagree) to 4 (strongly agree). Some samples of the questions are as follows: “I can accept psychological intervention in the form of self-help micro-videos.” “The content of the micro-videos and assignments is easy to understand.” “Micro-video psychological intervention is helpful for me.” The last one is an open question about your suggestions and opinions for our psychological intervention.

## 4. Statistical analysis

The analyses will be conducted based on the principle of intention-to-treat, meaning that all participants who have been randomized will be included in the analyses. In statistical tests, the SPSS version 23.0 software will be selected and used. The relevant data will be considered statistically significant if P < 0.05. The categorical variables are statistically described by the number of cases (percentage), and the chi-square analysis is used for intergroup comparison. Continuous variables are statistically described using mean ± standard deviation (M ± SD), and the independent sample *t*-test is used to make comparisons between the two groups. Also, repeated measures, such as ANOVA, are used to compare the degree of symptom improvement between the intervention group and the control group, and the interaction of group × time is analyzed. Missing data will be imputed using the estimation maximization (EM) method in SPSS version 23.0.

## 5. Discussion

This online trial will examine whether a self-help psychological intervention platform that helps alleviate depression and/or anxiety symptoms and enhance resilience will be effective in terms of clinical outcomes and applicability.

This study has several inherent limitations. First, there may be dropouts in either of the two groups. We will conduct a dropout analysis and a survey to examine the reasons why the dropouts withdrew from the study. Second, since we will only use self-report questionnaires instead of examiner-rating scales or formal diagnostic instruments, there may be a bias resulting from the participants knowing their group. In addition, the elderly and participants with severe symptoms may have some inconvenience when using their mobile phones for online measurement. We opted for self-rating because, theoretically, the measurement and intervention should be easily accessible and highly applicable. Third, we anticipate that younger people, more psychologically engaged individuals, or more motivated people will be attracted by this open recruitment strategy. Accordingly, we should be cautious when generalizing results about a certain type of population. Finally, using a waiting list trial design poses substantial limitations on the validity and universality of the results. Mild to moderate depression and anxiety symptoms have a tendency to heal on their own, and there is also a probability that the control group will also take various self-help measures, which would underestimate the intervention effect of our study.

Our study also has several strong points. Populations with depression and anxiety symptoms constitute a large population group in China. In contrast, there is limited access to psychological resources and limited capacity to provide psychological intervention for this group. It is essential to look for optimized technology that uses a standardized procedure and produces a positive effect. Compared with previous mental health indicators that involved diagnostic depression, anxiety, and other mental disorders, symptoms as inclusion criteria and psychological resilience index as a supplementary indicator were selected in our study to change the disease-oriented intervention mode into a health-oriented intervention mode, realizing precise mental health intervention (6). These technologies have the potential to evolve further and become available to many more people. As a non-stigmatizing and complementary psychological intervention resource to address, prevent, treat, and promote mental health, they have the prospects to produce sustainable and positive effects on people’s mental healthcare and, by extension, on society.

## Ethics statement

This study was approved by the Ethics Committee of Shanghai Mental Health Center (SMHC) (Approval Document Number: 2020-13) and registered on (Chinese Clinical Trial Registry) ChiCTR.org.cn (ChiCTR2100043725). All participants who voluntarily participate in this study shall provide the electronic informed consent.

## Author contributions

JQ contributed to the conception of the study and obtained funding for the trial as the primary investigator. WZhao and SC collaboratively developed the study design and prepared the manuscript. WZhao wrote the manuscript. SC did coordination work during the protocol implementation. JH and QZ were the research cooperators in the community. JT, JZ, YWu, SS, YWa, YS, YP, and WJ recruited participants from psychological clinics. WZhao, WJ, JT, QF, and RG developed the structure and content of the *Micro-Video Psychological Training Camp (MVPTC)* under the guidance of YWu and JQ. WZhang and JC gave important suggestions about the study design. All authors reviewed the manuscript and approved the final version.
